# Application of High-Performance Liquid Chromatography Coupled with Linear Ion Trap Quadrupole Orbitrap Mass Spectrometry for Qualitative and Quantitative Assessment of Shejin-Liyan Granule Supplements

**DOI:** 10.3390/molecules23040884

**Published:** 2018-04-11

**Authors:** Jifeng Gu, Weijun Wu, Mengwei Huang, Fen Long, Xinhua Liu, Yizhun Zhu

**Affiliations:** 1 Shanghai Key Laboratory of Bioactive Small Molecules, Department of Pharmacology, School of Pharmacy, Fudan University, Shanghai 201203, China; jifeng.gu@fdeent.org (J.G.); 13211030034@fudan.edu.cn (W.W.); 16211030050@fudan.edu.cn (M.H.); 13301030080@fudan.edu.cn (F.L.); liuxinhua@fudan.edu.cn (X.L.); 2 Department of pharmacy, Eye & ENT Hospital of Fudan University, Shanghai 200031, China; 3 State Key Laboratory of Quality Research in Chinese Medicine and School of Pharmacy, Macau University of Science and Technology, Macau, China

**Keywords:** Shejin-liyan Granule, HPLC-LTQ-Orbitrap MS, identification, quality assessment

## Abstract

A method for high-performance liquid chromatography coupled with linear ion trap quadrupole Orbitrap high-resolution mass spectrometry (HPLC-LTQ-Orbitrap MS) was developed and validated for the qualitative and quantitative assessment of Shejin-liyan Granule. According to the fragmentation mechanism and high-resolution MS data, 54 compounds, including fourteen isoflavones, eleven ligands, eight flavonoids, six physalins, six organic acids, four triterpenoid saponins, two xanthones, two alkaloids, and one licorice coumarin, were identified or tentatively characterized. In addition, ten of the representative compounds (matrine, galuteolin, tectoridin, iridin, arctiin, tectorigenin, glycyrrhizic acid, irigenin, arctigenin, and irisflorentin) were quantified using the validated HPLC-LTQ-Orbitrap MS method. The method validation showed a good linearity with coefficients of determination (r^2^) above 0.9914 for all analytes. The accuracy of the intra- and inter-day variation of the investigated compounds was 95.0–105.0%, and the precision values were less than 4.89%. The mean recoveries and reproducibilities of each analyte were 95.1–104.8%, with relative standard deviations below 4.91%. The method successfully quantified the ten compounds in Shejin-liyan Granule, and the results show that the method is accurate, sensitive, and reliable.

## 1. Introduction

Pharyngitis is an inflammation of the pharyngeal mucous membrane and submucous lymphoid tissues that affects people of all ages around the world. Patients often report pain and irritation in the throat. In certain cases, the illness is not caused by an infection; in such a setting, antibiotics may be the wrong choice for treatment or they may only offer a modest improvement in symptoms [1,2]. Traditional Chinese medicines have been successfully used to treat pharyngitis for thousands of years because of their variety of multi-target, therapeutic, and synergistic effects [1,3,4].

Shejin-liyan Granule (SJLYKL) is a traditional Chinese herbal medicine that has been used to treat pharyngitis in clinical practice. It consists of six herbs: Rhizoma Belamcandae, *Arctium lappa*, radix *Sophorae tonkinensis*, *Physalis alkekengi*, radix Platycodonis, and radix Glycyrrhizae. These chemicals, such as the isoflavones from Rhizoma Belamcandae [5], physalins from *Physalis alkekengi* [6], ligands from *Arctium lappa* [7], and alkaloids from *Sophorae tonkinensis* [8] have been proven to have good anti-inflammatory, antibacterial, and antioxidant activities [9,10,11]. SJLYKL is an organic combination of complex and diverse chemical constituents from these six herbs, and its anti-pharyngitis effect may be closely related to these compounds. Therefore, systematically analyzing the constituents of SJLYKL could provide an interpretation of the material basis for its pharmacological effects.

The complete profiles and quantities of the bioactive ingredients in SJLYKL are not well understood. Several studies have focused on identifying the components in each of the herbs included in SJLYKL [5,6,12,13,14]; however, no method has been developed to systematically analyze SJLYKL. Therefore, it is important to develop a systematic qualitative and quantitative evaluation method for the pharmacologically active compounds in SJLYKL.

Liquid chromatography-mass spectrometry (MS) has become increasingly common because of its high selectivity and sensitivity. The combination of a linear ion tap with a high-resolution Orbitrap analyzer has been used for qualitative and quantitative analyses in various applications, including bioactive compounds of traditional Chinese medicines, metabolites, and drug abuse [15,16,17]. In this study, we developed a simple and rapid method for the comprehensive qualitative and quantitative analyses of the major constituents of SJLYKL. This is the first time that a technique using high-performance liquid chromatography coupled with linear ion trap quadrupole Orbitrap high-resolution mass spectrometry (HPLC-LTQ-Orbitrap MS) has been applied to identify and quantify the components in SJLYKL.

## 2. Materials and Methods

### 2.1. Reagents and Materials

Chlorogenic acid, neochlorogenic acid, cryptochlorogenic acid, arctiin, arctigenin, tectoridin, tectorigenin, iridin, irisflorentin, irigenin, matrine, oxymatrine, liquiritin, isoliquiritin, liquiritigenin, isoliquiritigenin, and galuteolin were obtained from the Shanghai YuanYe Biotechnology Co., Ltd. (Shanghai, China) for use as references. Glycyrrhizic acid and glycyrrhetinic acid were purchased from China’s National Institute for the Control of Pharmaceutical and Biological Products (Beijing, China). The purity of the reference compounds was >98% based on high-performance liquid chromatography (HPLC) analysis. SJLYKL was prepared by the Shanghai Liantang pharmacy (Shanghai, China). Deionized water was purified using a Milli-Q system (Millipore, Bedford, MA, USA). Ammonium acetate (HPLC grade; purity ≥98.0%) and acetic acid (HPLC grade; purity ≥99.7%) were provided by ANPEL Laboratory Technologies (Shanghai, China). Chromatographic grade methanol and acetonitrile were purchased from Merck (Darmstadt, Germany).

### 2.2. Sample Preparation

SJLYKL (0.1 g) was accurately weighed and extracted with 10 mL of 75% methanol in an ultrasonic bath at room temperature. The weight loss was compensated by adding 75% methanol after extraction, and the extract was centrifuged at 17,000× *g* for 10 min. The resulting supernatant was diluted by a factor of 10 using pure water; then, a 10-μL aliquot of the supernatant was injected into the HPLC-LTQ-Orbitrap MS system for analysis. Samples that were above the upper limit of quantification were diluted and reanalyzed.

### 2.3. Chromatographic and Mass Conditions

Chromatographic separation was performed on a Dikma ODS C_18_ column (150 × 4.6 mm, 5 μm) maintained at 30 °C using a Dionex HPLC system (Thermo Fisher Scientific, Sunnyvale, CA, USA). The gradient elution was 30 min at a flow rate of 1.0 mL/min using solvent A (water with 0.1% acetic acid and 2 mM ammonium acetate in water) and solvent B (acetonitrile) as the mobile phase. The elution was run on the following schedule: 10–20% B at 0–5 min, 20–50% B at 5–20 min, 50–60% B at 20–21 min, 60–90% B at 21–25 min, 90% B at 25.1–30 min, 90–10% B at 30–30.1 min, and 10% B at 30.1–36 min.

For qualitative analysis, the Orbitrap resolution of the survey scan was set to 30,000 and that for the MSn scan was set to 15,000. The data-dependent MS2 scanning was performed to trigger fragmentation spectra of the target ions and to prevent repetition by the dynamic exclusion settings. Peaks were identified by comparison with those of the standards. For those peaks that did not correspond to the standards, a database including about 200 major compounds was established by collecting information from the literature on the six herbs in SJLYKL, including their names, formulas, accurate molecular weights, and MS2 information. The accurate masses of the additive ions, such as [M + H]^+^, [M + Na]^+^, [M − H]^−^, and [M + HCOO]^−^, were also calculated. The MS detection was performed in selected ion monitoring mode to quantify the ten compounds, including matrine, galuteolin, tectoridin, iridin, arctiin, tectorigenin, glycyrrhizic acid, irigenin, arctigenin, and irisflorentin.

### 2.4. Method Validation for Quantitative Analysis

Stock standard solutions of these ten compounds were separately prepared in methanol and kept at 4 °C. A mixed working solution was prepared before each use and diluted to the appropriate concentration to create calibration curves. The calibration curve of each compound was prepared using at least five different concentrations. The external standard method was constructed using the area with respect to known concentrations of the test compound (C, μg/mL) and weighting of reciprocal concentrations (1/C^2^). The limits of detection (LOD) under the present chromatographic conditions were determined at a signal-to-noise ratio of 3.

The intra-day and inter-day accuracies (deviation from the nominal concentration (%)) and precisions (relative standard deviation, RSD%) were analyzed at different concentrations on one day, and this experiment was repeated for three consecutive days.

To evaluate the recovery of the method, known amounts of these compounds were added to SJLYKL, and the samples were then quantified as described above. The recovery of each analyte was calculated according to the following equation:

(1) $$\mathrm{Recovery}\;\left(\%\right)=\frac{A_{\det}-A_{\mathrm{orig}}}{A_{\mathrm{spi}}}\times 100$$

where *A*_det_ is the total detected amount of each compound, *A*_orig_ is the original amount of each compound in SJLYKL, and *A*_spi_ is the spiked known amount of each component. The amount of the target compound was calculated using the corresponding calibration curve.

To investigate the repeatability, five samples from the same batch of SJLYKL were accurately weighed and treated as described above. The sample stability was assessed by analyzing SJLYKL samples stored at 4 °C, after 0, 4, 8, and 24 h.

## 3. Results and Discussions

### 3.1. Development of the Extraction Method

The factors that affect the extraction efficiency, including the extraction solvents, extraction method, and extraction times, were investigated to optimize the sample extraction efficiency. The ultrasonic bath extraction method was convenient and effective for the examined components; therefore, further experiments were performed using ultrasonic bath extraction. Several components, such as arctiin, arctigenin, tectoridin, and tectorigenin, were not completely extracted using pure methanol as a solvent; thus, different water-methanol ratios (0:100, 25:75, and 50:50, *v*/*v*) were screened. The yield of arctiin, arctigenin, tectoridin, and tectorigenin increased significantly when extractions were performed with 75% or 50% methanol, and fewer interfering peaks were found when 75% methanol was used. The duration of the extraction (30 min, 40 min, 50 min, or 60 min) was also investigated to optimize the extraction procedure. The optimal extraction of SJLYKL (0.1 g of powder) was obtained using 10 mL of 75% methanol in an ultrasonic water bath for 30 min.

### 3.2. Profiles of Ingredients in the SJLYKL Extract

**Figure 1:**
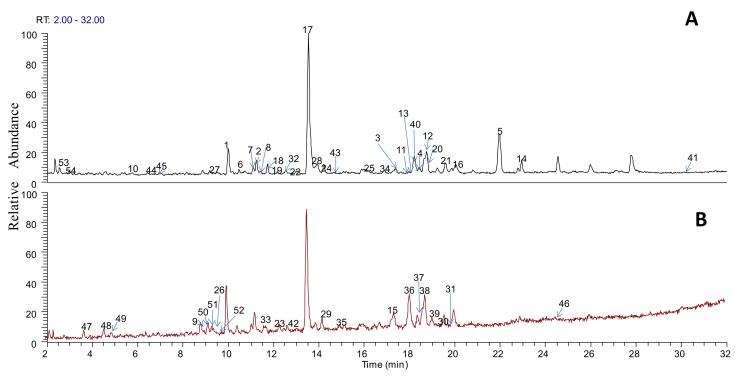
Base peak chromatogram of extract of Shejin-liyan Granule in positive mode (**A**) and negative mode (**B**).

In the analysis of the obtained chromatographic peaks by the HPLC-LTQ-Orbitrap MS method, we excluded the peaks which were the obtained parent accurate molecular weights without product ion. Then, to prevent misunderstanding caused by interference peaks, some peaks with an absolute intensity lower than 104 were removed. As a result, by comparing the information collected from the literature and standards with the data obtained by the HPLC-LTQ-Orbitrap MS method mass spectrometry, a total of 54 compounds were identified from the SJLYKL extract, including fourteen isoflavones, eleven ligands, eight flavonoids, six physalins, four triterpenoid saponins, six organic acids, two xanthones, two alkaloids, and one licorice coumarin (Table 1). The chemical structures of the 54 compounds are available online as Supplementary Materials. Typical peak chromatograms in positive and negative ion modes are shown in Figure 1.

Fourteen isoflavones, including seven isoflavone *O*-glucosides, four aglycones, and three isoflavones with methylenedioxy groups, were unambiguously or tentatively identified [18]. By comparing the retention times and the MS spectra of the SJLYKL extract with those of the standards, five peaks were unambiguously characterized as tectoridin (peak 1), iridin (peak 2), tectorigenin (peak 3), irigenin (peak 4), and irisflorentin (peak 5). Peaks 6, 7, 8, and 9 exhibited a high intensity [M + H − 162]^+^ ion, and were tentatively characterized as iristectorin B, iristectorin A, isoiridin, and 3,5-dimethoxyirisolone-4-*O*-glucoside, respectively, based on daughter ions [19]. Peak 10 showed a loss of 162 Da at *m*/*z* 463.1236 and an aglycon ion [M + H − 162 − 162] at *m*/*z* 301.0707; therefore, this compound was inferred as tectorigenin-7-*O*-glucosyl-4-*O*-glucoside [5]. Besides known aglycones, peaks 11 and 12 shared the same molecular ions and fragment pathways, and they were deduced as iristectorigenin A and iristectorigenin B, respectively, based on reported data [5]. Peaks 13 and 14 yielded diagnostic ions at [M + H − CH_3_]^+^ and [M + H − 2CH_3_ − CO]^+^, so they were tentatively characterized as noririsflorentin and dichotomitin, respectively [13].

Eleven of the detected ligands (except for peaks 15 and 16) generated adduct ions of [M + N_a_]^+^ or [M + NH_4_]^+^ in positive mode. By direct comparison with reference compounds, peaks 16 and 17 were readily identified as arctigenin and arctiin, respectively. Based on the accurate masses and literature data, peaks 15, 18, 19, 20, and 21 were identified as matairesinol, matairesinoside, lappaol H, lappaol A, and lappaol F, respectively [12,20]. Peaks 22, 23, 24, and 25 were of isomers with the adduct ions of [M + Na]^+^ at 577.2048 in positive mode, and they yielded fragment ions at [M + H − CH_3_]^+^ and [M + H − OCH_3_]^+^. These four peaks were ambiguously assigned as lappaol C, isolappaol C, lappaol E, and arctignan A, respectively [7,12].

Six flavonoids were identified by comparisons to reference standards: Liquiritin (peak 26), galuteolin (peak 27), isoliquiritin (peak 28), liquiritigenin (peak 29), isoliquiritigenin (peak 30), and formononetin (peak 31). Peaks 32 and 33 exhibited high-intensity [M + H − 132]^+^ ions and fragment ions formed by the successive loss of 162 Da, and were assigned as liquiritin apioside and isoliquiritin apioside, respectively.

Six physalins were detected. Peaks 34 and 35, with the same deprotonated molecular ion at *m*/*z* 543.1866 in negative ion mode, represent a pair of stereoisomers. Their parent and product ions were in agreement with physalin D and physalin D’, and the polarity of physalin D’ was stronger than that of physalin D; the compound with the shorter retention time should be physalin D’ [6]. Using the same method, the other two pairs of stereoisomers, peaks 36 and 37, and peaks 38 and 39, were identified as physalin F and physalin A, and physalin O and physalin L, respectively [6,13,21].

The four triterpene saponins were acidic saponins, and glycyrrhizic acid (peak 40) and glycyrrhetinic acid (peak 41) were identified by comparing their retention times and accurate masses with those of the standards. Peak 42 showed [M + H]^+^ ions at *m*/*z* 839.4070, which is 16 Da greater than that of glycyrrhizic acid, so it could be assigned to licorice saponin G2 [14]. In positive ion mode, peak 43 gave [M + H]^+^ at *m*/*z* 985.4645, [M − 162 + H]^+^ at 823.4116, and [M − 2 × 162 − H_2_O + H]^+^ at *m*/*z* 615.3897. Based on the cleavage patterns, which were similar to those of glycyrrhizic acid, the compound was presumed to be licorice saponin A3 [14,22]. 

Peaks 44 and 45 showed [M + H]^+^ ions at *m*/*z* 423.0925 in positive ion mode; this was similar to the accurate masses of mangiferin and isomangiferin ([M + H]^+^, 423.0924). The positive MS^2^ spectrum of *m*/*z* 423.09 showed fragment ions at 405.0819, 333.0607, and 303.0501, which correspond to losses of 18, 90, and 120 Da, indicating that the fragment ions were [M + H − H_2_O]^+^, [M + H − C_3_H_6_O_3_]^+^, and [M + H − C_4_H_8_O_4_]^+^, resulting from cross-ring cleavages in the sugar moiety. This result is consistent with the cleavage pattern of C-glycosidic xanthone and patterns in the literature [13]. Therefore, peaks 44 and 45 were tentatively identified as mangiferin and isomangiferin.

Peak 46 was tentatively characterized as glycyrol, a licorice coumarin, because of its [M − H]^−^ ion at *m*/*z* 365.1028 and the fragmentation at *m*/*z* 307.0244 ([M − H − C_4_H_10_]^−^) and *m*/*z* 295.0244 ([M − H − C_5_H_10_]^−^), which were the same as those of glycyrol [23].

Organic acids and alkaloids were identified by comparing their retention times and accurate masses with those of the standards. Peaks 47, 48, and 49 were identified as chlorogenic acid, neochlorogenic acid, and cryptochlorogenic acid, respectively, by comparison with reference compounds. Similarly, because of available standards, peaks 50, 51, 52, 53, and 54 were unequivocally identified as isochlorogenic acid B, isochlorogenic acid A, sochlorogenic acid C, oxymatrine, and matrine, respectively.

### 3.3. Quantitative Analysis

**Figure 2:**
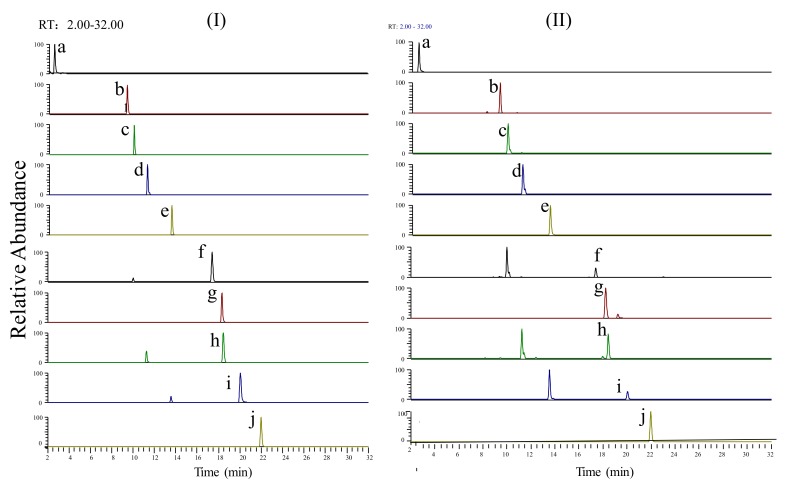
Extracted ion chromatograms of the (**I**) reference standards and (**II**) SJLYKL sample: (**a**) matrine; (**b**) galuteolin; (**c**) tectoridin; (**d**) iridin; (**e**) arctiin; (**f**) tectorigenin; (**g**) glycyrrhizic acid; (**h**) irigenin; (**i**) arctigenin; (**j**) irisflorentin.

**Figure 3:**
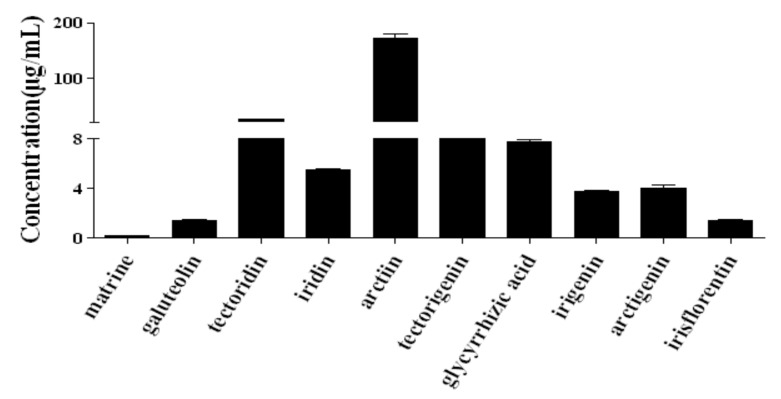
Content determination results of the ten compounds in Shejin-liyan Granule.

Extracted ion chromatograms of the standard sample and SJLYKL samples are shown in Figure 2. The results of regression analysis and LOD values for the ten compounds are shown in Table 2. All calibration curves showed good linearity (r^2^ > 0.9914) between the peak area and concentration. The accuracy of the intra- and inter-day variation of these investigated compounds was 95.0–105.0% and the precision values were less than 5.0% (Table 3). The results of the recovery and repeatability test are shown in Table 4. The recovery of all analytes was 95.1–104.8%, and the RSD values of the repeatability results were less than 4.94%. The sample solution was stable for 24 h at 4 °C (RSD < 4.31%, data not shown). Then, the proposed method was applied to analyze ten compounds in five SJLYKL samples. The identified levels of these compounds are summarized in Figure 3. Arctiin was present in the highest concentration, followed by tectoridin. These two components provide good anti-inflammatory effects [24,25] and may be active ingredients in SJLYKL. Therefore, these compounds could be marker compounds for quality control of SJLYKL.

## 4. Conclusions

In this study, a simple, accurate, and reliable HPLC-LTQ-Orbitrap MS method was established to qualitatively determine the 54 components of SJLYKL. The method successfully quantified ten major components in five batches of SJLYKL samples. This novel approach was useful to identify constituents and control the quality of SJLYKL. These results offer useful information for understanding the material basis of the therapeutic effects of SJLYKL and for its clinical application.

## Figures and Tables

**Table 1:** Identified compounds of Shejin-liyan Granule by HPLC-LTQ-Orbitrap method.

No.	Retention Time (min)	Experimental Mass (*m/z*)	Theoretical Mass (*m/z*)	Ion Type	Identification Compound	MS/MS Fragments
1	9.99	463.1237	463.124	[M + H]^+^	tectoridin	301.0706, 286.0472
2	11.23	523.1447	523.1342	[M + H]^+^	iridin	361.0919
3	17.38	301.0706	301.0711	[M + H]^+^	tectorigenin	286.0475
4	18.43	361.0921	361.0923	[M + H]^+^	irigenin	346.0685
5	21.97	387.109	387.109	[M + H]^+^	irisflorentin	357.0606
6	10.6	493.1342	493.134	[M + H]^+^	iristectorin B	331.0815, 316.0579
7	11.1	493.1342	493.134	[M + H]^+^	iristectorin A	331.0815, 316.0579
8	11.46	523.1447	523.1452	[M + H]^+^	isoiridin	523.1447, 361.0919
9	12.78	535.1446	535.1452	[M + H]^+^	3,5-dimethoxyirisolone-4-*O*-d-glucoside	373.0919, 358.0685
10	6.16	625.1765	625.1767	[M + H]^+^	tectorigenin-7-*O*-glucosyl-4-*O*-glucoside	463.1236, 301.0707, 286.0473
11	17.98	331.0815	331.0818	[M + H]^+^	iristectorigenin A	316.0582, 301.0347
12	18.5	331.0813	331.0818	[M + H]^+^	iristectorigenin B	316.0582, 301.0348
13	18.12	373.0918	373.0923	[M + H]^+^	noririsflorentin	358.0687
14	22.37	359.0761	359.0764	[M + H]^+^	dichotomitin	326.0424
15	17.28	357.1342	357.1338	[M − H]^−^	matairesinol	342.0380, 327.0149
16	20.02	373.1643	373.1651	[M + H]^+^	arctigenin	355.1539
17	13.53	552.2443	552.2445	[M + NH_4_]^+^	arctiin	373.1647, 355.1542
18	11.72	538.2286	538.2288	[M + NH_4_]^+^	matairesinoside	359.1491
19	11.99	773.2786	773.2785	[M + Na]^+^	lappaol H	755.2684, 725.2574
20	18.8	559.1938	559.1944	[M + Na]^+^	lappaol A	397.1259
21	19.6	732.302	732.302	[M + NH_4_]^+^	lappaol F	531.2014
22	12.8	577.2048	577.205	[M + Na]^+^	lappaol C/isolappaol C/lappaol E/arctignan A	559.1945, 562.1801
23	13.94	577.2048	577.205	[M + Na]^+^	lappaol C/isolappaol C/lappaol E/arctignan A	559.1945, 562.1804
24	14.2	577.2046	577.205	[M + Na]^+^	lappaol C/isolappaol C/lappaol E/arctignan A	559.1947, 562.1801
25	16.1	577.2042	577.205	[M + Na]^+^	lappaol C/isolappaol C/lappaol E/arctignan A	559.1944, 562.1802
26	9.18	417.1187	417.1186	[M − H]^−^	liquiritin	255.0653
27	9.34	449.1083	449.1084	[M + H]^+^	galuteolin	287.0555
28	12.34	417.1187	417.1186	[M − H]^−^	isoliquiritin	255.0653
29	14.24	255.0662	255.0657	[M − H]^−^	liquiritigenin	153.0917
30	19.08	255.0663	255.0657	[M − H]^−^	isoliquiritigenin	153.0918
31	19.8	267.0653	267.0657	[M − H]^−^	formononetin	252.0431
32	8.86	549.1608	549.1608	[M − H]^−^	liquiritin apioside	417.1192, 255.0665
33	11.84	549.1608	549.1608	[M − H]^−^	isoliquiritin apioside	417.1192, 255.0666
34	12.97	543.1833	543.1866	[M − H]^−^	physalin D’	525.1763, 515.1919
35	15.22	543.1833	543.1866	[M − H]^−^	physalin D	525.1763, 515.1919
36	17.92	525.1759	525.1761	[M − H]^−^	physalin F	507.1663, 497.1819
37	18.18	525.176	525.1761	[M − H]^−^	physalin A	507.1663, 497.1819
38	18.53	527.1915	527.1917	[M − H]^−^	physalin O	509.1815
39	19.01	527.1916	527.1917	[M − H]^−^	physalin L	509.1815
40	18.22	823.4116	823.4116	[M + H]^+^	glycyrrhizic acid	647.3791, 471.3467, 453.3363
41	30.26	471.3464	471.3474	[M + H]^+^	glycyrrhetinic acid	425.3416
42	16.89	839.407	839.4065	[M + H]^+^	licorice-saponin G2	663.3747, 469.3312, 451.3210
43	14.53	985.4645	985.4644	[M + H]^+^	licorice saponin A3	823.4116, 647.3806, 615.3897, 453.3366
44	6.44	423.0923	423.0924	[M + H]^+^	mangiferin	405.0819, 333.0607, 303.0501
45	6.77	423.0923	423.0924	[M + H]^+^	isomangiferin	405.0819, 333.0607, 303.0501
46	24.63	365.1028	365.1025	[M − H]^−^	glycyrol	307.0244, 295.0244
47	3.7	353.0876	353.0873	[M − H]^−^	chlorogenic acid	191.0565, 179.0352
48	4.58	353.0876	353.0873	[M − H]^−^	neochlorogenic acid	191.0565, 179.0352
49	4.97	353.0876	353.0873	[M − H]^−^	cryptochlorogenic acid	191.0565, 179.0352
50	8.94	515.1188	515.119	[M − H]^−^	isochlorogenic acid B	353.0878
51	9.14	515.1193	515.119	[M − H]^−^	isochlorogenic acid A	353.087
52	9.42	515.1191	515.119	[M − H]^−^	isochlorogenic acid C	353.0876
53	2.49	249.1962	249.1967	[M + H]^+^	matrine	148.1122
54	3.14	265.1913	265.1916	[M + H]^+^	oxymatrine	247.1810, 205.1339

**Table 2:** Regression equation, correlation coefficients, and LOD of the ten compounds.

Compound	Regression Equation	r^2^	Liner Range (μg/mL)	LOD (μg/mL)
matrine	*y* = 46117 + 5066555*x*	0.9914	0.020–2.5	0.002
galuteolin	*y* = 1550 + 1112359*x*	0.9985	0.023–3.0	0.002
tectoridin	*y* = 30165 + 1743884*x*	0.9988	0.027–3.5	0.002
iridin	*y* = 4672 + 1112803*x*	0.9921	0.016–2.0	0.002
arctiin	*y* = 7991 + 2241923*x*	0.9988	0.195–25	0.002
tectorigenin	*y* = 34605 + 11608484*x*	0.9987	0.023–3.0	0.002
glycyrrhizic acid	*y* = 67891 + 2468972*x*	0.9954	0.023–3.0	0.002
irigenin	*y* = 1793 + 447209*x*	0.9972	0.025–3.2	0.002
arctigenin	*y* = 7894 + 1314941*x*	0.9994	0.02–2.5	0.002
irisflorentin	*y* = 54529 + 50506549*x*	0.9989	0.016–2.0	0.0005

**Table 3:** Intra- and inter-day precision and accuracy of the ten compounds.

Compound	Concentration (μg/mL)	Intra-Day				Inter-Day			
		Mean	±	SD	Accuracy (Bias %)	Mean	±	SD	Accuracy (Bias %)
matrine	0.040	0.038	±	0.0014	−5.00	0.0398	±	0.002	−0.50
	0.640	0.651	±	0.022	1.70	0.669	±	0.015	4.50
	1.875	1.782	±	0.087	−5.00	1.822	±	0.082	−2.80
galuteolin	0.046	0.0438	±	0.002	−4.80	0.044	±	0.002	−4.30
	0.736	0.769	±	0.028	4.50	0.755	±	0.006	2.60
	2.250	2.21	±	0.061	−1.80	2.34	±	0.059	4.00
tectoridin	0.054	0.052	±	0.001	−3.70	0.0516	±	0.001	−4.40
	0.864	0.811	±	0.009	−6.10	0.827	±	0.010	−4.30
	2.625	2.63	±	0.102	0.20	2.56	±	0.079	−2.50
iridin	0.032	0.033	±	0.001	1.90	0.0307	±	0.0004	−4.10
	0.512	0.531	±	0.005	3.70	0.533	±	0.002	4.10
	1.500	1.55	±	0.069	3.30	1.543	±	0.035	2.90
arctiin	0.400	0.42	±	0.014	5.00	0.38	±	0.013	−5.00
	6.400	6.303	±	0.046	−1.50	6.21	±	0.054	−3.00
	18.750	17.9	±	0.456	−4.50	17.89	±	0.432	−4.60
tectorigenin	0.046	0.0496	±	0.0007	7.80	0.0464	±	0.0004	0.90
	0.094	0.091	±	0.002	−2.90	0.09	±	0.003	−4.00
	2.250	2.15	±	0.056	−4.40	2.36	±	0.081	4.90
glycyrrhizic acid	0.046	0.045	±	0.002	−2.20	0.0438	±	0.002	−4.80
	0.736	0.769	±	0.009	4.50	0.772	±	0.009	4.90
	2.250	2.32	±	0.060	3.10	2.33	±	0.065	3.60
irigenin	0.050	0.052	±	0.0008	4.00	0.0522	±	0.0004	4.40
	0.800	0.797	±	0.002	−0.40	0.809	±	0.001	1.10
	2.400	2.32	±	0.016	−3.30	2.35	±	0.018	−2.10
arctigenin	0.040	0.038	±	0.001	−5.00	0.0391	±	0.001	−2.20
	0.640	0.653	±	0.005	2.00	0.652	±	0.006	1.90
	1.875	1.86	±	0.053	−0.80	1.85	±	0.071	−1.30
irisflorentin	0.032	0.0336	±	0.0003	5.00	0.031	±	0.0004	−3.10
	0.500	0.512	±	0.001	2.40	0.513	±	0.001	2.60
	1.500	1.465	±	0.013	−2.30	1.457	±	0.015	−2.90

**Table 4:** Recovery and repeatability of these ten compounds (*n* = 3).

Compound.	Recovery					Reproducibility (μg/mL)			
	Spiked Amount (μg)	Dectected Amount (μg)			Accuracy (Bias %)	Mean	±	SD	RSD
matrine	1.0	1.027	±	0.04	4.80	0.19	±	0.003	1.67
galuteolin	7.3	7.28	±	0.25	−0.10	1.39	±	0.03	2.27
tectoridin	125.8	119.6	±	5.85	−4.90	24.33	±	0.53	2.18
iridin	27.3	26.9	±	1.23	−1.50	5.68	±	0.25	4.40
arctiin	863.5	862.1	±	31.56	−0.20	169.60	±	2.50	1.47
tectorigenin	56.7	55.6	±	1.52	−1.90	11.00	±	0.54	4.91
glycyrrhizic acid	38.8	37.1	±	0.64	−4.50	7.49	±	0.37	4.94
irigenin	19.0	18.2	±	0.19	−4.30	3.92	±	0.15	3.83
arctigenin	20.3	19.5	±	0.75	−3.90	4.25	±	0.19	4.47
irisflorentin	7.2	6.9	±	0.26	−3.80	1.37	±	0.07	4.91

## Supplementary Materials

The chemical structures of the 54 compounds are available online.
