# Supplementary material for: Application of High-Performance Liquid Chromatography Coupled with Linear Ion Trap Quadrupole Orbitrap Mass Spectrometry for Qualitative and Quantitative Assessment of Shejin-Liyan Granule Supplements
Source: Molecules. 2018 Apr 11;23(4):884. doi: 10.3390/molecules23040884 (PMC6017834; doi:10.3390/molecules23040884)
Supplement: Supplementary file 1 [file molecules-23-00884-s001.pdf]

Supplementary Materials:

The chemical structures of the 54 compounds were characterized as follow:

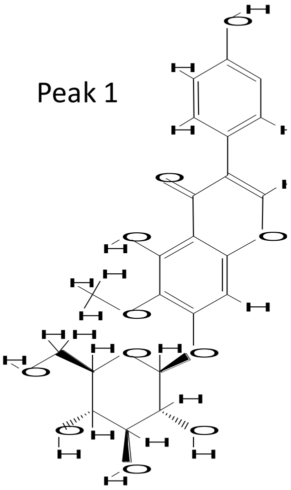

Peak 1

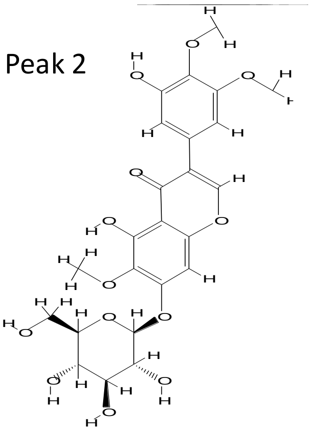

Peak 2

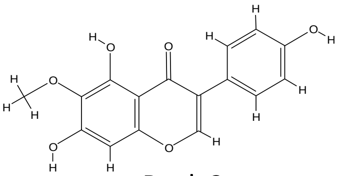

Peak 3

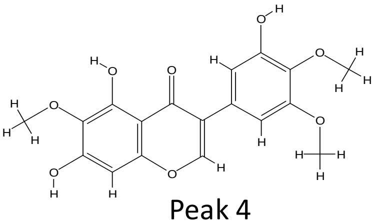

Peak 4

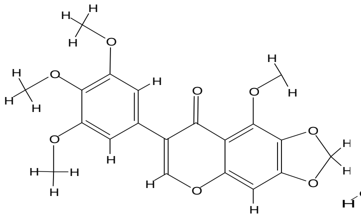

Peak 5

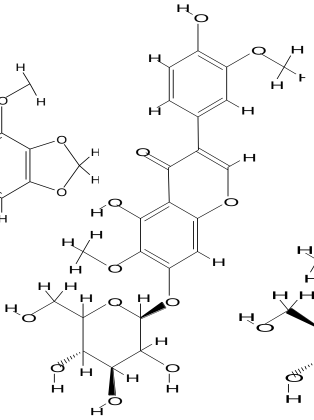

Peak 6

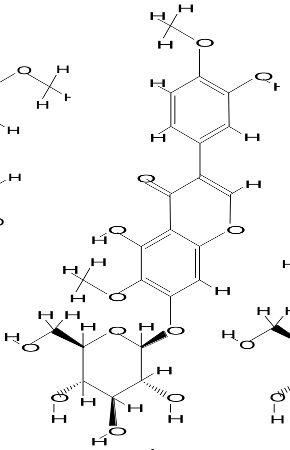

Peak 7

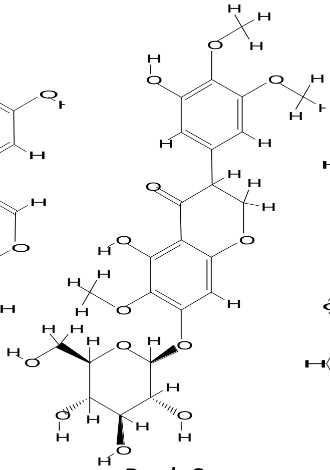

Peak 8

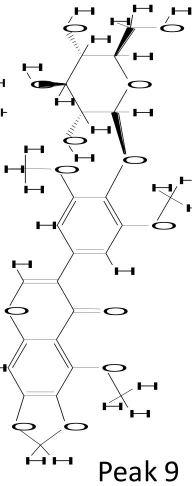

Peak 9

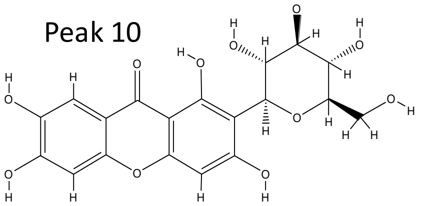

Peak 10

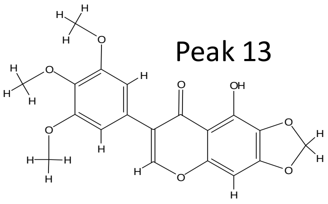

Peak 13

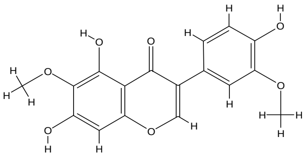

Peak 12

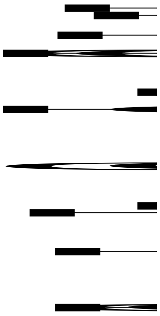

Peak 11

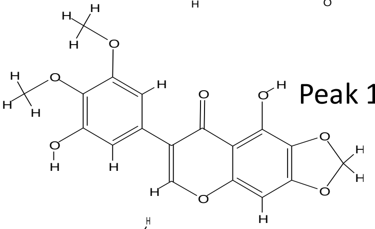

Peak 14

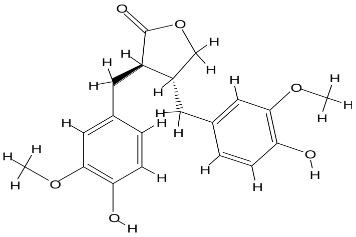

Peak 15

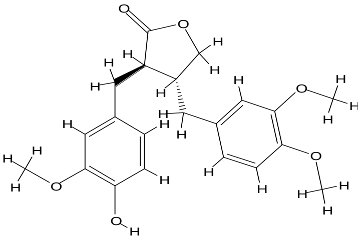

Peak 16

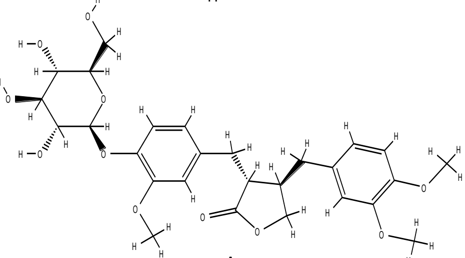

Peak 17

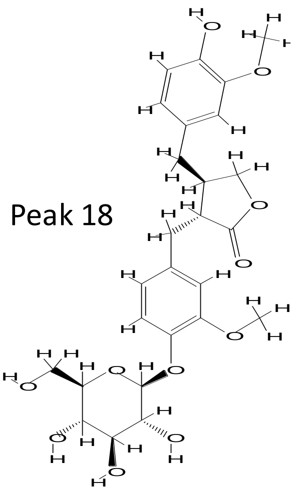

Peak 18

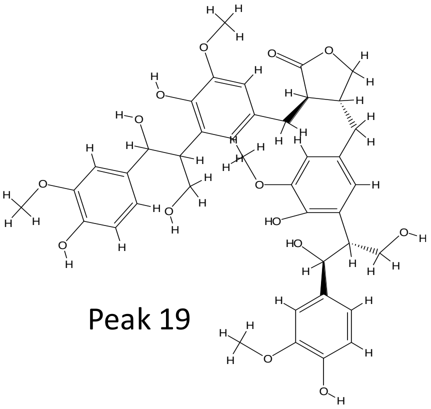

Peak 19

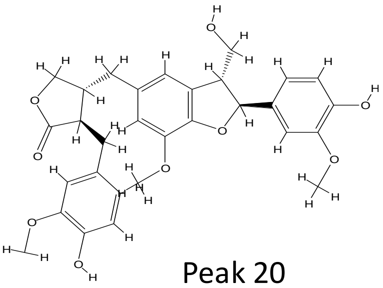

Peak 20

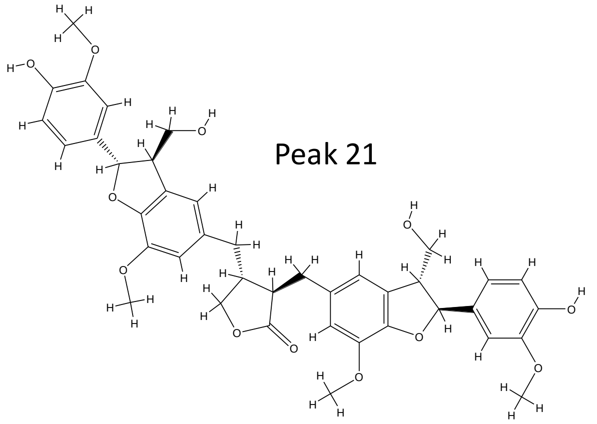

Peak 21

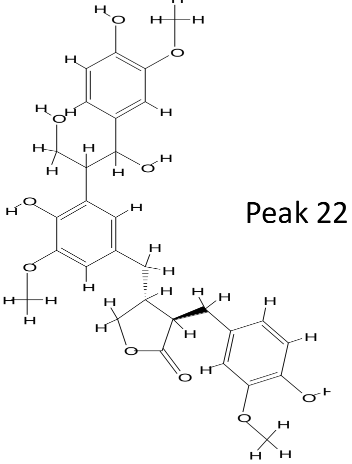

Peak 22

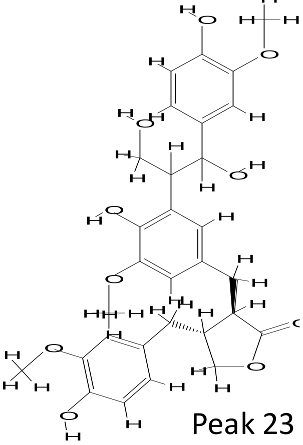

Peak 23

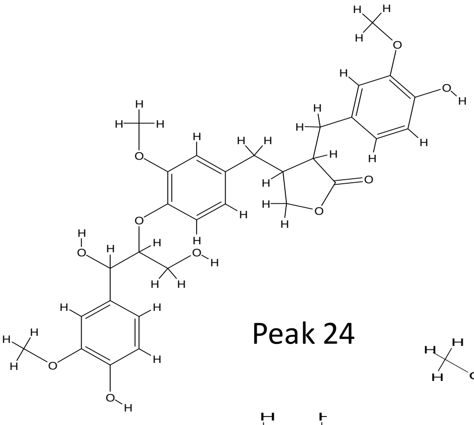

Peak 24

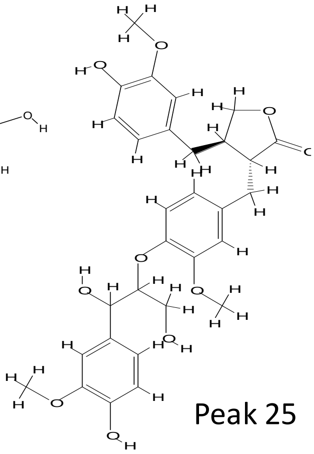

Peak 25

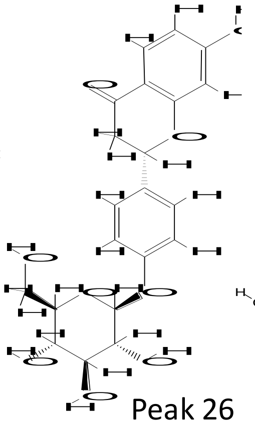

Peak 26

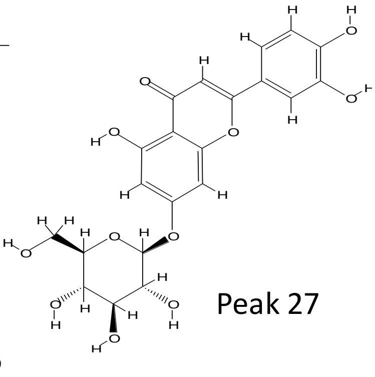

Peak 27

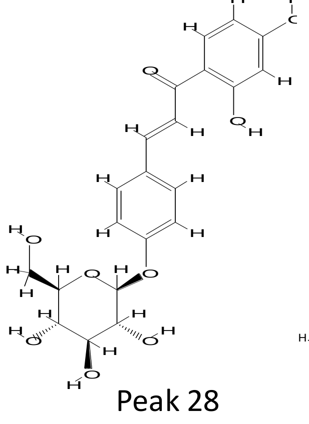

Peak 28

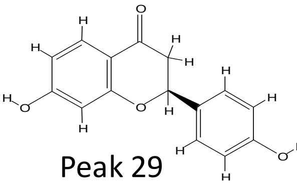

Peak 29

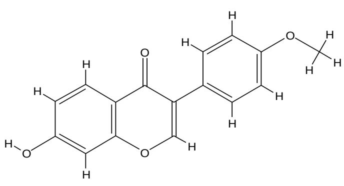

Peak 31

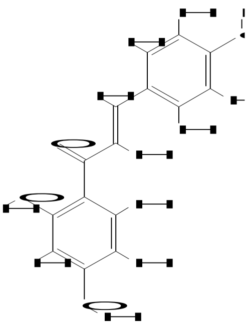

Peak 30

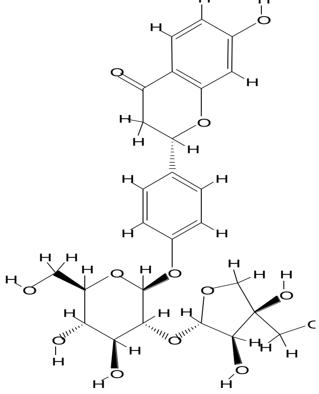

Peak 32

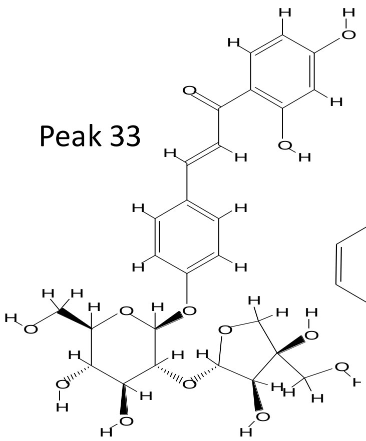
Peak 33

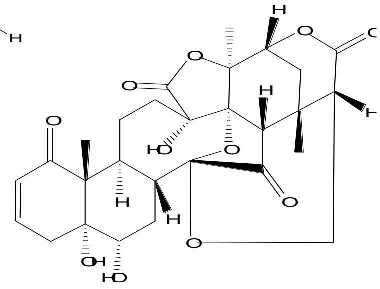
Peak 34

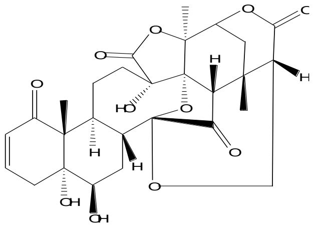
Peak 35

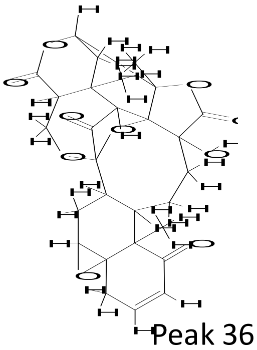
Peak 36

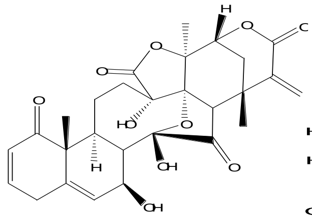
Peak 37

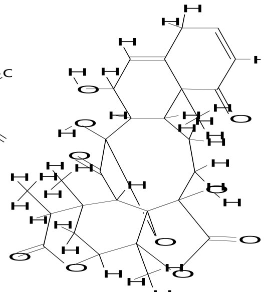
Peak 38

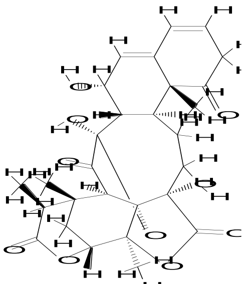
Peak 39

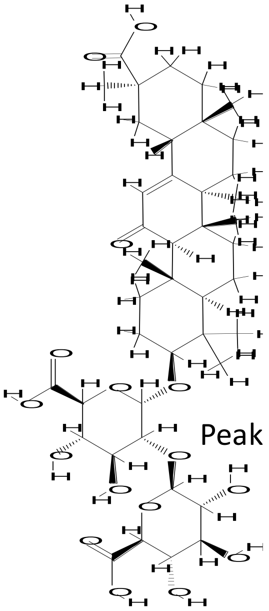
Peak 40

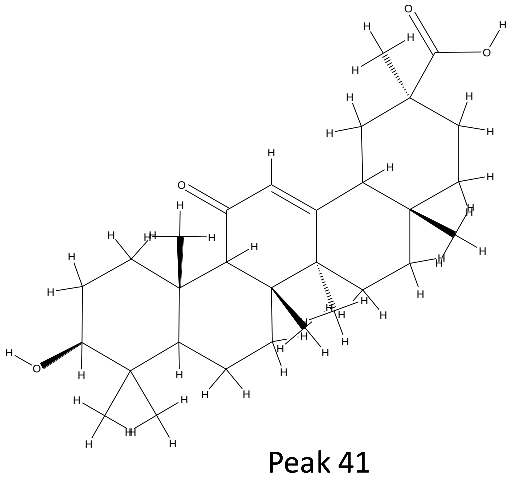
Peak 41

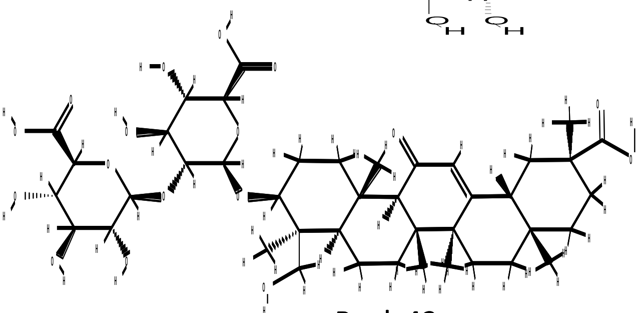
Peak 42

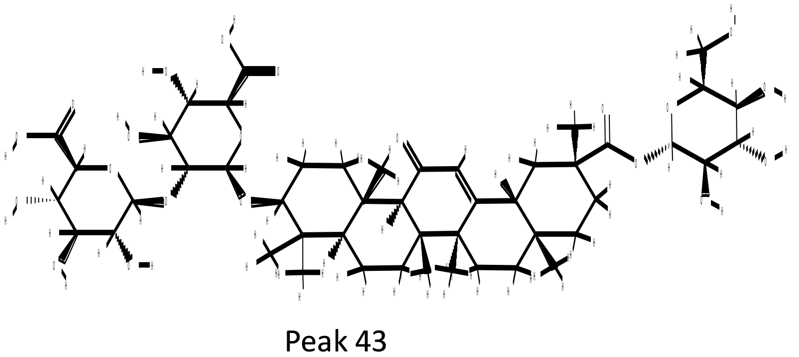
Peak 43

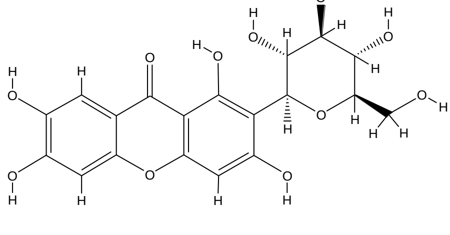
Peak 44

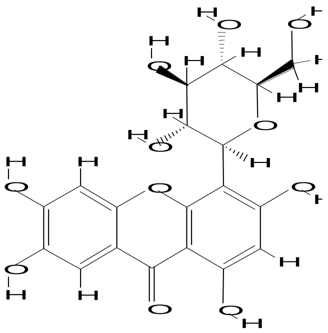

Peak 45

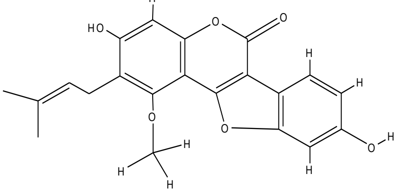

Peak 46

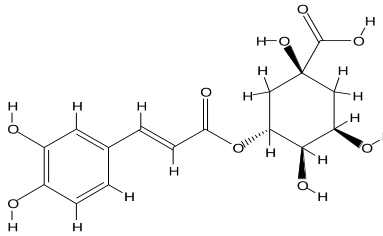

Peak 47

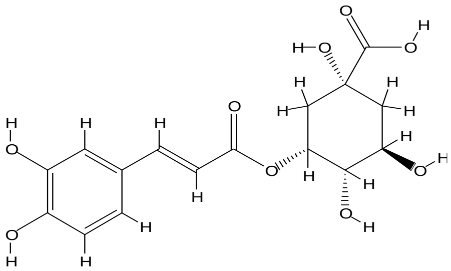

Peak 48

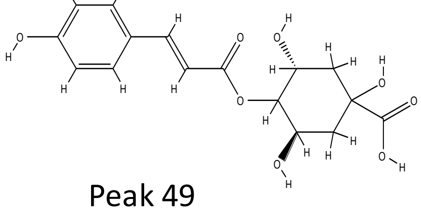

Peak 49

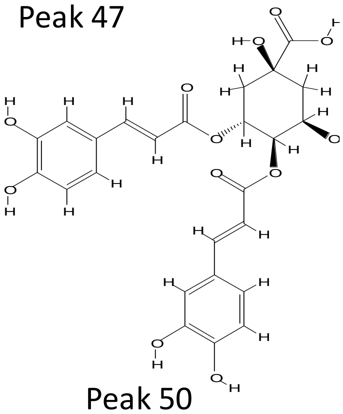

Peak 50

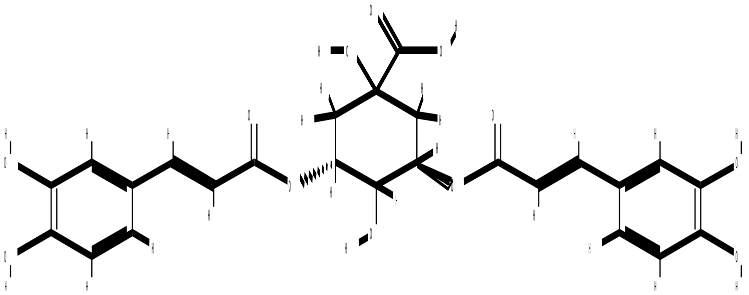

Peak 51

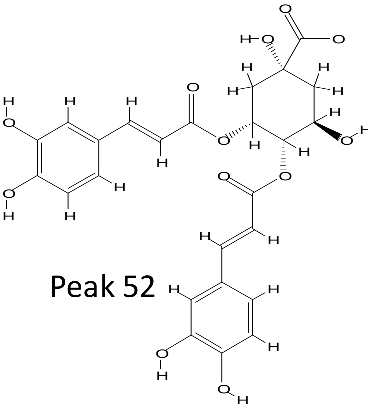

Peak 52

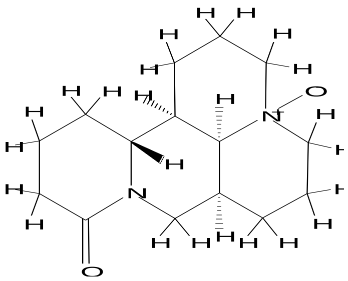

Peak 53

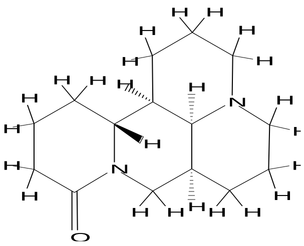

Peak 54
